# A Patient-focused Information Design Intervention to Support the Minor Traumatic Brain Injuries (mTBI) Choosing Wisely Canada Recommendation

**DOI:** 10.7759/cureus.5877

**Published:** 2019-10-09

**Authors:** Shawn Dowling, Heather Hair, Denise Boudreau, Daniel Grigat, Christopher Rice, Karen B Born, Stephanie VandenBerg

**Affiliations:** 1 Emergency Medicine, Physician Learning Program, Calgary, CAN; 2 Emergency Strategic Clinical Network, Alberta Health Services, Calgary, CAN; 3 Continuing Medical Education and Professional Development, University of Calgary, Calgary, CAN; 4 Epidemiology and Public Health, University of Toronto, Toronto, CAN; 5 Emergency Medicine, University of Calgary, Calgary, CAN

**Keywords:** head injury, choosing wisely, patient education, patient-centered care, minor traumatic brain injuries (mtbi)

## Abstract

Introduction

The first Choosing Wisely Canada (CWC) recommendation for Emergency Medicine states: “Don’t order CT head scans in adults and children who have suffered minor head injuries (unless positive for a validated head injury clinical decision rule)”. In order to provide patients with information on the risks and benefits of computed tomography (CT) scans in minor traumatic brain injuries (mTBI) and to encourage discussions between patients and their doctor, we designed a patient-focused mTBI infographic for the emergency department (ED).

Methods

Stakeholders worked with content experts to co-design the infographic, which was posted in two emergency department (ED) waiting rooms. A survey was administered to evaluate whether the infographic influenced patient beliefs about the risks and benefits of CT scans and to gauge patient willingness to have a discussion with their doctor about the necessity of a scan.

Results

One hundred fifteen patients completed the survey. Prior to participating, 38% of patients thought a CT after an mTBI was always a good idea and 60% thought it was sometimes a good idea. After viewing the poster, 87% of respondents stated they better understood when a CT scan may be appropriate, 93% felt they better understood the risks of CT scans, and 76% understood that their doctor can often rule out serious illness without a CT scan. Only 19% of patients still felt that a CT was always necessary after an mTBI.

Conclusions

The mTBI infographic changed patient perceptions regarding the need for CT scans and increased awareness of the indications and risks of CT scans. This study demonstrates that targeted patient education materials can help support CWC recommendations.

## Introduction

Choosing Wisely Canada (CWC) is an international initiative that aims to reduce unnecessary tests, treatments and procedures by engaging physicians and patients in conversations [1]. One of the recommendations in the CWC list for Emergency Medicine: Ten Things Physicians and Patients Should Question includes ‘Don’t order CT head scans in adults and children who have suffered minor head injuries (unless positive for a validated head injury clinical decision rule)’ [2]. Within the Canadian context, the most well-validated and widely known clinical decision rule (CDR) for mTBI is the Canadian CT head rule (CCHR) developed by Stiell et al. [3-4]. However, a study by Lin et al. exploring emergency physicians’ behavior and knowledge concerning CWC recommendations found that CT scans for minor traumatic brain injury (mTBI) was identified as the most commonly ordered low-value test [5]. In this study, the most commonly cited reason for ordering the CT for mTBI was perceived patient or family expectations, although the extent of patients' expectations of a CT scan and how they were communicated is unclear. 

Effective communication between health care providers and patients is central to health education and shared decision-making. This is especially critical in the emergency department (ED) setting where patient health and education depends on accurate and easy-to-understand information to facilitate a timely diagnosis, patient-centered discussions around management, and appropriate resource allocation [6]. When adequate information is not provided, patient uncertainty may result in misunderstandings, therapy non-compliance, increased use of health resources, and/or worse health outcomes [7-9]. Conveying health information from clinician to patient is a critical step in the patient-provider relationship within an ED and has many inherent challenges in a chaotic and unpredictable ED setting [7]. Traditionally, information regarding patient education has been offered after interaction with health care providers, and evidence suggests patients only retain about 20% of instructions given verbally by clinicians [10]; therefore, it may be reasonable and beneficial to introduce health information for common ED presentations prior to the patient-clinician encounter. This not only empowers the patient by allowing them to participate in their health care, but leads to informed discussions around diagnosis, further testing, and future care [11-12].

Shifting patient expectations and conversations about related risks, harms, and benefits can be challenging [13]. There is a growing body of evidence that up to 30% of health care is unnecessary and adds no value to patient care [14]. Patient expectations and preferences may influence care practices. In a presentation by Bansback et al., patients had a significant reduction in requesting or expecting low back pain imaging after reviewing the CWC patient pamphlets (Abstract: Bansback N, Chiu J, Kerr S, McCracken R, Forster B: Reducing Imaging Tests for Low Back Pain: Can Patients Choose Wisely? Presented at the American College of Rheumatology/American Reproductive Health Professionals (ACR/ARHP) Annual Meeting, Washington, DC 2016. http://acrabstracts.org/abstract/reducing-imaging-tests-for-low-back-pain-can-patients-choose-wisely). As a result, helping patients and clinicians engage in informed conversations and the shared decision may lead to a reduction in unnecessary treatment or testing.

This study aimed to evaluate the use of a patient infographic on perceptions of the risks and benefits of a CT scan for mTBI in two Calgary, Alberta EDs. The specific study objectives were to quantify the change in perception as a result of interaction with the intervention (infographic) measured using pre- and post-intervention self-reported answers to questions regarding the need for a CT in mTBI. Secondary objectives included baseline patient perceptions of when a CT scan is necessary, risks associated with CTs, and patient willingness to engage in a discussion with their physician about the risks and benefits of medical imaging.

## Materials and methods

Alberta Health Services is the province-wide health system in Alberta, Canada. The health system has developed Strategic Clinical Networks™ on priority areas focused on quality improvement. The Emergency Strategic Clinical Networks™ (ESCN) mandate includes developing and supporting a system-wide approach to the delivery of emergency care for Albertans that is appropriate, patient-focused, timely, safe, and aligned with quality standards.

Infographic development

The ESCN, partnering with the University of Calgary Department of Emergency Medicine and the Mount Royal University Department of Information Design, created a patient-centered infographic to better inform the public on the risks and benefits of CT scans and encourage shared decision-making between patient and doctor [15].

The content of the infographic was selected to align with CWC recommendations on CT scans for mTBI and was developed in collaboration with a team of emergency physicians. After the infographic was designed, it was presented to two patient focus groups for user testing. The infographic was revised and refined based on feedback from the patient focus groups. Major changes based on patient feedback included removing wording about “high” risk criteria, as patients interpreted this is indicating a probability of 90% - 100% that neurosurgery was required. The final design was evaluated against the International Patient Decision Aid Standards checklist [16].

**Figure 1:**
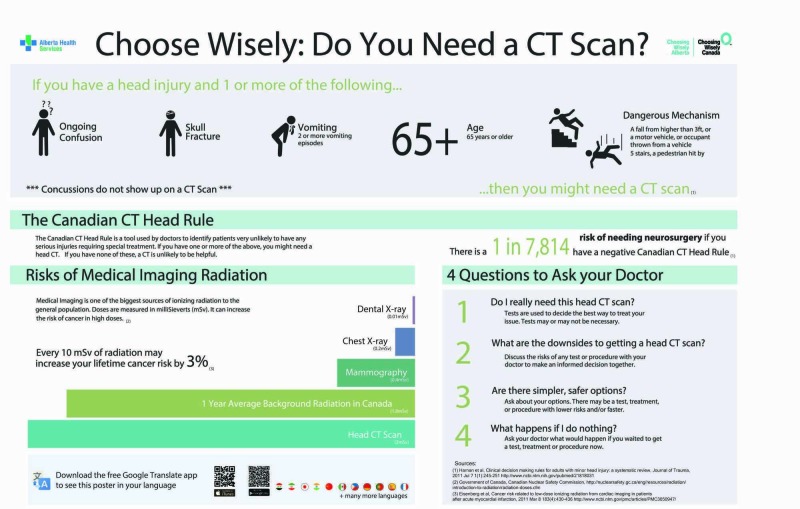
Minor traumatic brain injury infographic Harnan et al. [4]; Radiation Doses (Canadian Nuclear Safety Commission) [17]; Eisenberg et al. [18] Google Translate App (Google LLC, Mountain View, CA) CT: computed tomography

The key elements of the infographic (Figure 1) included: 1) the CCHR explained in such a way that the key messages were understandable by patients; 2) pros and cons of CT scans; and 3) guidance on how to have a discussion with your doctor about CTs for mTBI. The infographic also included a “Google translate” quick response (QR) code to translate the infographic into different languages.

The infographic was posted in high visibility areas in two local ED waiting areas - Foothills Medical Center and Peter Lougheed Hospital - which see an average of 215 mTBI presentations a month [19]. The Foothills Medical Center is the quaternary care facility and a Level 1 trauma center in Calgary, and the Peter Lougheed Hospital is a community hospital that has a diverse socioeconomic patient population. Investigators worked with key stakeholders (departmental leadership and site operations) for approval to display the infographic and ensure it met local standards, such as infection prevention and control standards. This project received approval from the Conjoint Health Research Ethics Board at the University of Calgary (REB16-2050). 

Study methodology

To evaluate our study objectives, we developed a short mixed-methods survey consisting of one demographic question, six questions regarding patient perceptions on the risks and benefits of CT scans, and two open-ended questions on improving the infographic design and other comments patients wished to share. Participants were asked to complete the survey after reviewing the mTBI infographic. The survey was piloted by six emergency physicians prior to use to ensure readability and clarity. The survey was designed to evaluate whether the infographic influenced patient beliefs about the risks and benefits of CT scans and patient willingness to engage in a discussion with their doctor. Quantitative data were aggregated and analyzed using descriptive statistics in Microsoft® Office Excel (Microsoft® Corp., Redmond, WA).

Hospital volunteers approached participants based on convenience while they were in the ED waiting area. The study was open to all patients waiting and not just those specifically with an mTBI. Informed consent was obtained and participants were given the time to review the mTBI infographic, at which time the participants were able to complete the survey using one of two methods to collect the data: 1) the volunteer approached patients and family members in the ED waiting, and if agreeable, consent was obtained and the survey was completed on the study team’s electronic device or 2) study participants in the ED waiting area were able to scan the QR on the infographic code using their own personal device to consent and complete the survey.

## Results

Over a 56-day period, 115 surveys were completed. Of the 115 participants who consented and completed the survey, 32% were aged 18 - 34, 51% were aged 35 - 64, and 17% were aged 65 or older.

**Figure 2:**
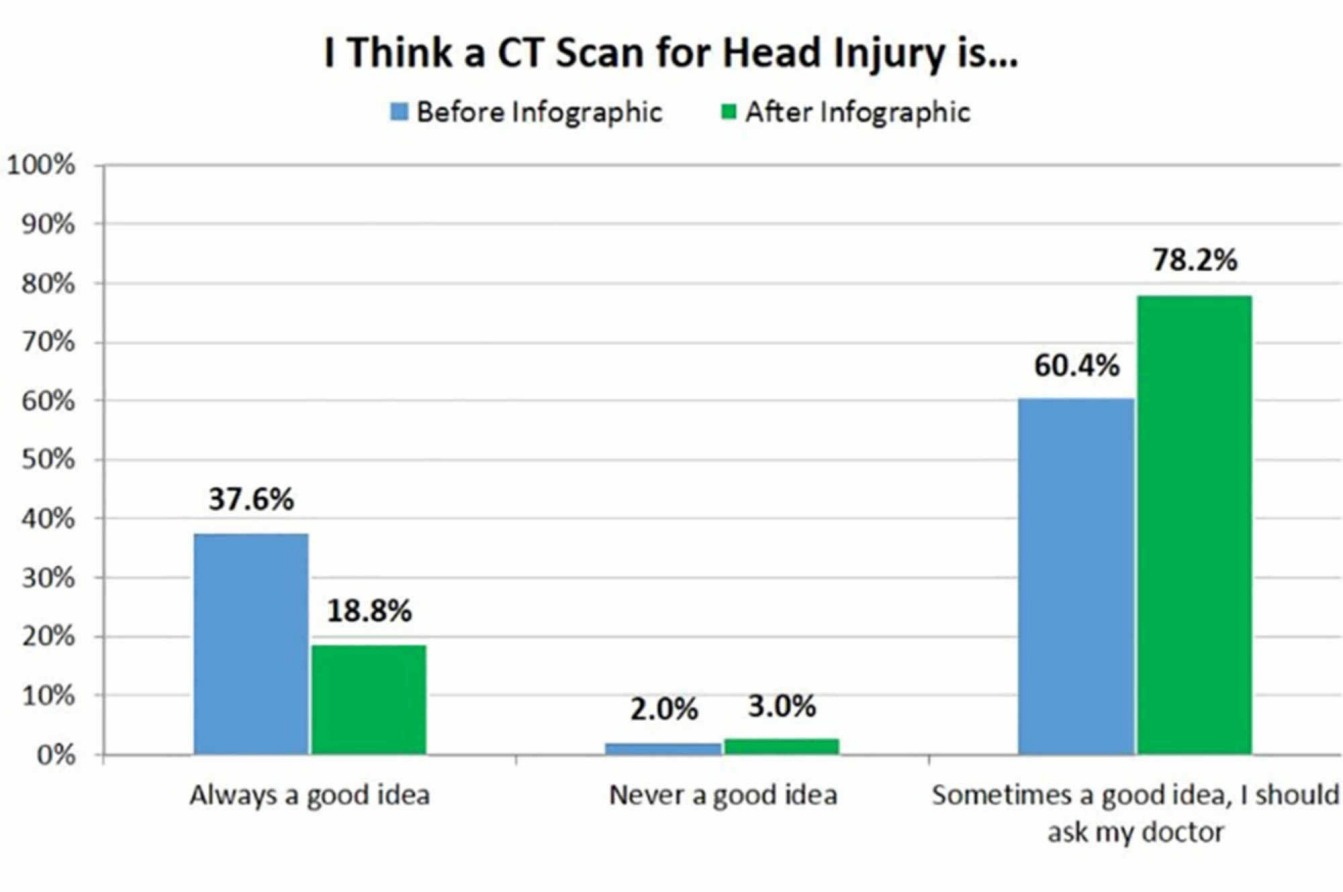
Respondents' perception of when computed tomography (CT) is necessary after a head injury

Prior to reading the infographic, 38% of respondents thought that a CT scan was always a good idea following a head injury, and 60% thought it required discussion with their doctor. Following exposure to the infographic, the percentage of respondents who thought a CT scan was always a good idea dropped to 19%, and the percentage who thought it required discussion with their doctor increased to 78% (Chi-squared value: 7.773, p-value: 0.005) (Figure 2).

**Figure 3:**
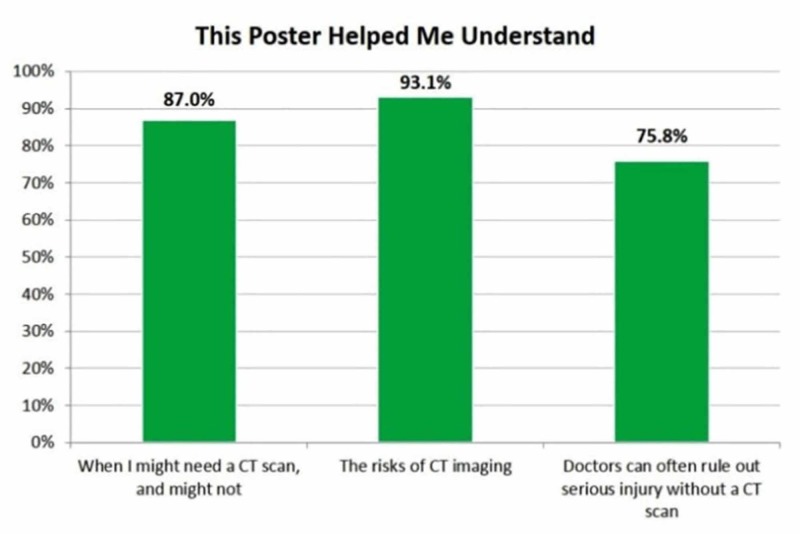
Respondents' understanding of the infographic CT: computed tomography

**Figure 4:**
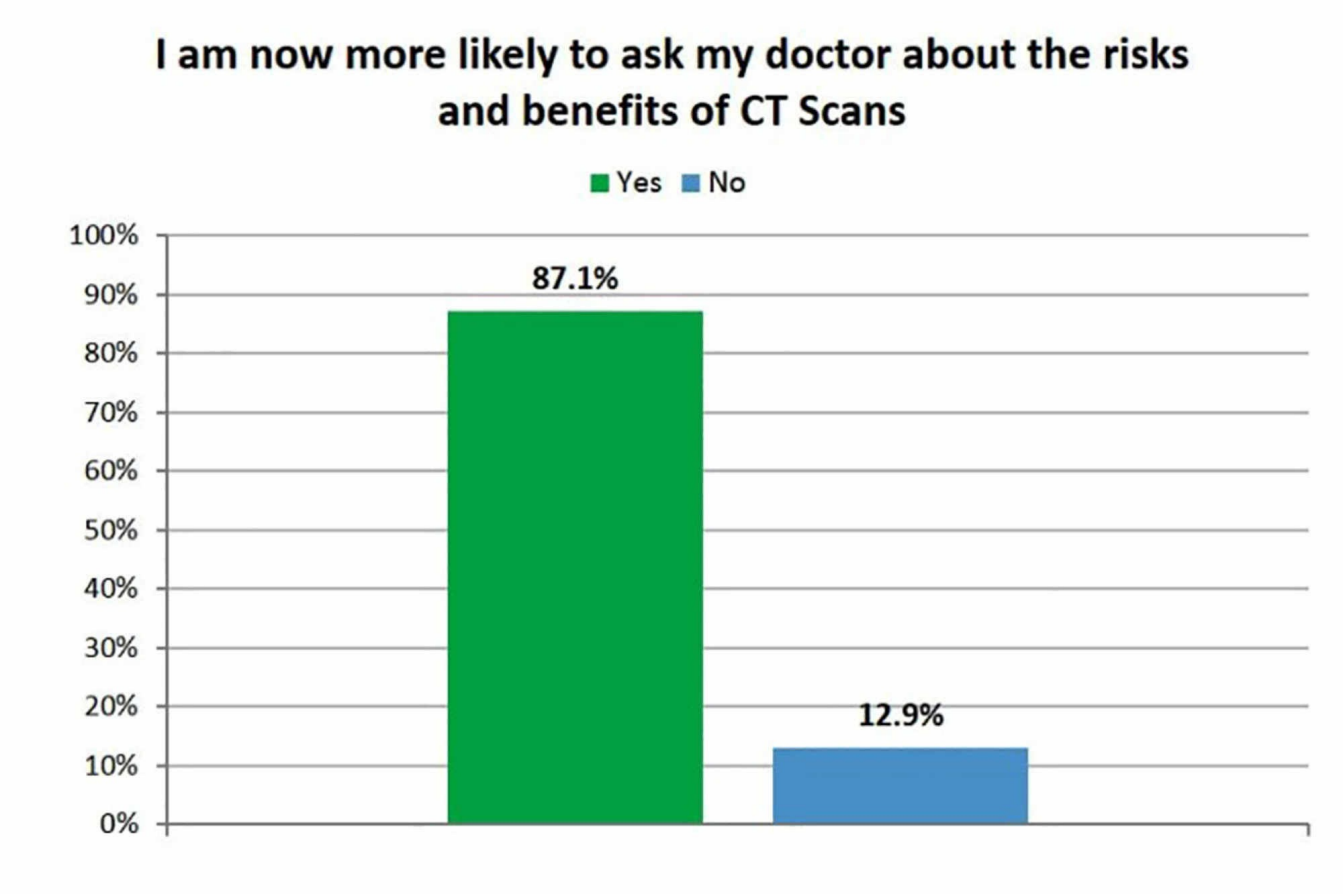
Respondents' willingness to discuss the risks and benefits of computed tomography (CT) scans

Following a review of the infographic, 87% of respondents stated that they better understood when they might or might not need a CT scan, 93% reported that they better understood the risks of CT imaging, and 76% stated they understood that doctors can often rule out serious injury without a CT scan (Figure 3). Eighty-seven percent of respondents reported that they were now more likely to discuss the risks and benefits of CT scans with their doctor (Figure 4).

When the results were analyzed by age, 97% of respondents aged 18 - 34 reported that they understood when they might need a CT scan compared to 77% of respondents aged 65 or older (Chi-squared value: 3.063, p-value: 0.080). No differences in response by age range were found to be statistically significant. 

Twelve respondents provided comments in the free text section of the survey (Table 1). Four provided commentary on the content of the infographic, such as: “Risk of radiation exposure is an important question that the public needs to be aware of. The issue of head injury and the warning signs needing further investigation is also valuable information.” Three respondents commented in approval of the infographic design, such as: “I love the graph!!! That stuck out most as a visual comparison,” while two respondents offered suggestions for improvement: “Not impressed with poster size.” One respondent asked about remuneration for the study (none was provided), one respondent asked if doctors follow the infographic guidelines, and one respondent raised concerns about access to CT scans and wait times.

**Table 1:** Survey Respondents' Free Text Comments CT: computed tomography

Comment Theme	Comment Frequency
Commented on the content of the infographic (e.g., risk of radiation)	N = 4
Commented on the design of the infographic (e.g., use of graphs)	N = 3
Suggestions for improvement (e.g., increase poster size)	N = 2
Asked for remuneration	N = 1
Asked if doctors follow these guidelines	N = 1
Raised concerns about CT access and wait times	N = 1

## Discussion

This study supports the use of infographics to provide concise information on a complex topic to increase patient understanding and education on a specific health care concern. Usability tests and informed design principles with respect to the intended target audience and key messages reveal how to create effective infographics and should be referred to by anyone considering their use. The novel design of patient education material has the potential to improve patient understanding of key health information, allowing them to participate in their own care and decision-making. Future areas of research may include the continued use of information design partnered with technology (video and interactive tablets) to increase content usability and improve knowledge translation. Since this project was completed, the infographic has been introduced to 16 EDs across Alberta and modified for digital television displays in hospitals and primary care offices.

Promoting better conversations between patients and physicians to facilitate informed decisions is a core aim of the CWC campaigns worldwide [20]. Shared decision-making, supported by decision aids (like infographics) can help increase health literacy and patient knowledge to reduce the overuse of medical interventions [21].

Limitations

How people respond to information about their health is directly related to their health literacy and how health information is presented [22]. This study did not attempt to measure baseline health literacy using a standardized scoring system; therefore, it is unclear whether this population’s ability to comprehend health information is different from other populations, or if the patients who consented to the study had a different level of health literacy. However, patients presenting to an ED generally represent a heterogeneous group of individuals with various levels of health literacy by the nature of its mandate to provide universal healthcare at any time to anyone. Because of logistical challenges, we were unable to assess baseline knowledge and perceptions prior to reviewing the infographic; as a result, participants were only asked to complete the survey after reviewing the infographic. 

The study was designed to minimize ED flow disruption and maximize patient participation. We chose to administer the survey before the patient had been assessed by a clinician; however, the patient may have had the opportunity to learn about their condition and associated testing prior to participating in the study, even if they had not been told a probable diagnosis. By excluding patients with high acuity/distress, language barriers (such as English as a second language), or altered mental status, we limited the generalizability of our study. Because of patient confidentiality and resource issues, hospital volunteers approached any patients in the waiting room, not just those with an mTBI. Finally, we were unable to assess whether perceptions and expectations actually resulted in a change of physician ordering behaviour (reduction in CT scans being performed). Future studies are needed to address this patient-level outcome.

## Conclusions

The mTBI infographic changed patient perceptions regarding the need for CT scans, improved understanding of indications for CT, and the risks of CT scans in the setting of mTBI. This study demonstrates that targeted patient education materials can help support CWC recommendations, and inform patients and the public about the harms of unnecessary tests.
